# Preference and willingness to pay for reproductive health services among adults in Urban–Rural transition settings of a developing country: evidence from a cross-sectional study in a rural district of Hanoi, Vietnam

**DOI:** 10.1186/s12913-023-10207-1

**Published:** 2023-11-02

**Authors:** Nguyen Thao Thi Nguyen, Long Hoang Nguyen, Tham Thi Nguyen, Linh Gia Vu, Thuc Minh Thi Vu, Minh Ngoc Le Vu, Giang Thu Vu, Carl A. Latkin, Cyrus S. H. Ho, Roger C. M. Ho

**Affiliations:** 1 Department of Obstetrics and Gynecology, Duke University Medical Center, Durham, NC 27710 USA; 2 Department of Public Health Sciences, Karolinska Institutet, Stockholm, 17177 Sweden; 3 Institute for Global Health Innovations, Duy Tan University, Da Nang, 550000 Vietnam; 4 Faculty of Nursing, Duy Tan University, Da Nang, 550000 Vietnam; 5 Institute of Health Economics and Technology, Hanoi, 100000 Vietnam; 6 Center of Excellence in Evidence-Based Medicine, Nguyen Tat Thanh University, Ho Chi Minh City, Vietnam; 7 Bloomberg School of Public Health, Johns Hopkins University, Baltimore, MD 21205 USA; 8 Department of Psychological Medicine, Yong Loo Lin School of Medicine, National University of Singapore, Singapore, Singapore; 9 Institute for Health Innovation and Technology (iHealthtech), National University of Singapore, Singapore, Singapore

**Keywords:** Willingness to pay, Reproductive health services, WTP, RHS, Vietnam

## Abstract

**Background:**

Since the introduction of fee-for-service models in public hospitals and the legalization of private health services in Vietnam in 1989, the price of reproductive health services has risen. These changes have exacerbated inequities in accessing reproductive health services. This study examines potential disparities in willingness to pay for reproductive health services among adults in a rural district of Hanoi.

**Methods:**

A cross-sectional study was conducted at 9 communes in Thanh Oai district, a rural district of Hanoi, Vietnam, in July 2019. Face-to-face interviews were conducted using a structured questionnaire to collect self-reported data. The contingent valuation was used to examine willingness to pay for reproductive health services with a starting price of 2 million VND (~ US$86.2, July 2019 exchange rate), which is the average price of all RHS in public facilities in Vietnam. Multiple Logistic regression and Multiple Interval regression models were used to identify factors associated with willingness to pay and the amount that people were willing to pay for reproductive health services.

**Results:**

Among 883 participants, this study found 59.1% of them willing to pay for reproductive health services at an average maximum amount of US$36.2, significantly less than the current average price of US$86.2. Occupation, number of sex partners, perception towards the necessity of reproductive health services, and prior use of reproductive health services were found to significantly influence willingness to pay for reproductive health services, while age, income level, gender, occupation, perception towards the necessity of reproductive health services and prior use of reproductive health services were reportedly correlated with the amount participants were willing to pay for reproductive health services.

**Conclusion:**

Lower willingness to pay for reproductive health services compared to the current prices (US$36.2 vs. US$86.2) is likely related to an overall low awareness of the necessity of reproductive health services, and future education campaigns should specifically target those from lower-income backgrounds. Financial subsidization should also be provided, especially for those from the low-income group, to ensure equitable access to reproductive health services. Given the heterogeneity of reproductive health services, further studies should examine the willingness to pay for each type of service independently.

**Supplementary Information:**

The online version contains supplementary material available at 10.1186/s12913-023-10207-1.

## Background

Since the introduction of economic modifications in 1986, Vietnam’s economy has shifted from a centrally planned to a market economy. Its health services underwent a similar transition in 1989 with the introduction of fee-for-service models in public hospitals and the legalization of private health services [1]. Despite its financial barriers to low-income patients, private health services have become increasingly popular [2]. To counter these changes, the Vietnamese government instituted the National Strategy for Reproductive Health Care between 2001–2010, which aimed to provide universal low-cost reproductive health services (RHS) including family planning, antenatal care, and treatment of sexually transmitted infections [3]. One of the ways in which the country has expanded RHS to ensure access to low-income people is through local commune health stations, where local residents can receive subsidized or free services [4]. Though private health insurance is available, the country has also expanded its public program in the past three decades to subsidize medical costs for vulnerable groups including the poor, ethnic minorities, children, and the elderly [5].

As a result of these efforts and other factors, Vietnam has seen an improvement in the population’s overall reproductive health. Maternal mortality, for example, has decreased from 68 per 100,000 live births in 2000 to 43 per 100,000 in 2017 [6]. Antenatal care utilization has also increased from 68.6% in 2000 to 95.9% in 2014. Similarly, more women have utilized skilled staff during labor—from approximately 70% in 2000 to 90% in 2014 [7]. However, those improvements have not been equally distributed across all demographics. Despite the country’s efforts to expand access to RHS, certain demographics including higher economic status, higher education, urban residence, and Kinh ethnic majority continue to be associated with greater utilization of RHS [7–9]. The measurement of preferences for reproductive health services in both men and women is crucial, especially in countries going through a transition stage, such as Vietnam. Despite the commonly held belief that reproductive health services are geared toward women, it is important to acknowledge that reproductive health issues are not limited to females alone. Men's preferences and attitudes can have a significant impact on reproductive health outcomes for both genders [10]. In Vietnam, where traditional gender roles persist, and taboos surrounding candid discussions on sensitive issues exist, incorporating both men and women in the study would offer a more accurate reflection of reproductive health preferences and needs [11]. As such, the participation of both genders in the study is critical to developing effective reproductive health policies in Vietnam and other developing countries.

In light of these inequities in access and increasing privatization of the healthcare infrastructure, further research is required to assess the financial barriers to RHS. Prior studies in Vietnam have reported on willingness to pay (WTP) for various individual RHS, including WTP for hepatitis B virus (HBV) and human papillomavirus (HPV) vaccination. These studies found that the average price participants were willing to pay was half and two-thirds of the actual price for the HBV and HPV vaccines, respectively, highlighting the need for financial subsidization to improve access, especially for low-income people [12, 13]. However, similar analyses have not been performed for other important RHS including family planning, antenatal care (ANC), and treatment of sexually transmitted diseases. As such, this study aims to further elucidate WTP for RHS to identify barriers to care among adults with a focus on those living in a rural district of Hanoi. With the increasing privatization of healthcare in low- and middle-income countries [14] and a knowledge gap regarding WTP for RHS in those settings, this study aims to create a framework to guide future evaluations in similar settings outside of Vietnam as well.

## Methods

### Study setting and participants

We conducted a cross-sectional study in Thanh Oai district, Hanoi, Vietnam, in July 2019. Particularly, Thanh Oai is a rural district located in the south of Vietnam's capital, about 20 km from Hanoi city. In this study, we purposively selected 9 communes in Thanh Oai district to recruit participants, including Bich Hoa, Kim An, Lien Chau, Thanh Cao, Thanh Mai, Thanh Van, Kim Bai, Xuan Duong, and Tam Hung.

Subjects recruited into the study need characteristics consistent with selection criteria, including 1) Being 18 years old or older; 2) Agreeing to participate in the study, and 3) Having no health problems affecting the ability to perceive and respond to the survey. Only participants who met all the selection criteria were invited into the study.

### Sample and sampling

In this study, we use a convenience sampling method. The sample size was calculated using the formula to estimate the percentage of participants willing to pay for RHS with α = 0.05, and the percentage of participants willing to pay for RHS was 0.5 (since this is the first study on willingness to pay for RHS, we choose *p* = 0.5), relative error = 0.05. To prevent incomplete responses or dropout, 15% of the sample size was added to the sample size, resulting in a total of 884 participants who were invited to participate in the study. At the end of data collection, 890 participants enrolled in this study. In which, there were 883 participants completed the questionnaire (completion rate was 99.2%). The number of questionnaires that have been investigated in each region is presented in Appendix 1.

### Variables and instruments

Face-to-face interviews were conducted by the medical staff of Hanoi Medical University. This study used a structured questionnaire to collect self-reported data. These consisted of four major components: 1) demographics, 2) health status and health behaviors, 3) demand for RHS, and 4) willingness to pay for RHS. Participants were selected using convenience sampling.

### Outcomes

**Fig. 1:**
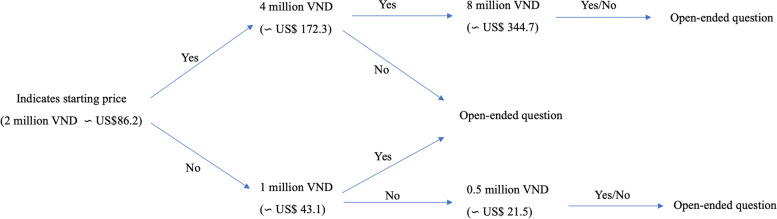
The bidding process *(Unit: 1 $US = 23,210 VND, July 19, 2019 exchange rate)*

To determine the willingness of the patients to pay, a contingent valuation (CV) approach was adopted in this study. The double-bounded dichotomous choice (DBDC) questions supported by an open-ended (OE) question were utilized. By using consecutive questions, the method attempts to mirror the behavior of consumers within a regular market [15]. This technique is more effective in providing estimations of WTP compared to utilizing a single question alone [16]. The starting price of RHS is 2 million VND (~ US$86.2, July 2019 exchange rate), which is the average price of all RHS in public facilities in Vietnam. Participants were initially asked, “Are you willing to pay 2 million VND per taking care of reproductive health service?” The price doubled or halved depending on the participant’s answer. The question was repeated until the last price reached four times higher or four times lower than the initial price (see Fig. 1). Finally, subjects were asked about the maximum price that they were willing to pay for RHS.

All values will be presented in US$ (July 19, 2019 exchange rate [17]).

### Predictors

Socioeconomic variables included were age group (young adults ages 18–30, middle-aged adults ages 31–45, or older adults ages greater than 45); sex; education level (less than high school, high school, or greater than high school); marital status; occupation; health insurance status (both public and private health insurance); and household income by quintile. In Vietnam, health insurance has two types: public and private. In terms of public health insurance, there are 2 types: compulsory and voluntary, people are required to buy compulsory health insurance and voluntary health insurance is optional but has more services. Public health insurance is not for profit, organized by the State. Regarding private health insurance, there are many service packages according to the price, with many different services with better service, medical examination, and treatment with modern and faster facilities.

The health status variables included body mass index (BMI), number of chronic diseases, current tobacco use, and number of sex partners. Participants were also asked to indicate the necessity of RHS (necessary, neutral, or unnecessary); their desire for RHS; and their use of RHS in the past.

### Data analysis

Collected data were entered into Epidata 3.1 and processed by Stata 16.0. All the entries were rechecked for possible data entry errors. Chi-square testing was used to assess differences in nominal variables.

According to the model used in the contingent valuation method, the value Y_i_ is considered as the willingness to pay (WTP) to RHS on an individual with attributes represented by X_i_ [18].

$${\mathrm{Y}}_{\mathrm{i}}={\mathrm{X}}_{\mathrm{i}}\upbeta + {\upvarepsilon }_{\mathrm{i}}$$

The normal distribution of εi with a Mean = 0. As we observe the respondents' willingness to pay indirectly through their answers to contingent valuation questions, we can determine that the value of Yi falls between the range of Yi1 and Yi2. The contribution of each individual's likelihood is shown as:

$$\mathrm{Pr}({\mathrm{Y}}_{\mathrm{i}}1\le {\mathrm{Y}}_{\mathrm{i}}\le {\mathrm{Y}}_{\mathrm{i}}2=\mathrm{Pr}({\mathrm{Y}}_{\mathrm{i}}1<{\mathrm{X}}_{\mathrm{i}}\upbeta <{\mathrm{Y}}_{\mathrm{i}}2)$$

If the available data is right-censored, indicating that some patients did not agree to pay the initial cost, which resulted in the absence of WTP information for the double bid and uncertainty regarding the upper limit, then the likelihood contribution is represented as $\mathrm{Pr}({\mathrm{X}}_{\mathrm{i}}\upbeta + {\upvarepsilon }_{\mathrm{i}} \le {\mathrm{Y}}_{\mathrm{i}}2)$.  

Similarly, if the data is left-censored, implying that some individuals were willing to pay the initial price, leading to a lack of WTP data for the half bid and uncertainty about the lower limit, then the likelihood contribution is represented as $\mathrm{Pr}({\mathrm{Y}}_{\mathrm{i}}1 \le {\mathrm{X}}_{\mathrm{i}}\upbeta + {\upvarepsilon }_{\mathrm{i}})$.  

Both the DBDC method and OE follow-up questions were utilized in this study. Normally, the WTP of each respondent was identified as left- or right-censored data based on their responses to WTP bids. However, the DBDC-OE method used in this study allowed for the combination of both uncensored and censored data. For example, suppose a customer was willing to pay US$ 100 for RHS, and then said “Yes” to a subsequent bid of US$ 200 for the same RHS, and they gave a maximum WTP for an RHS value of US$250 for the OE question. In the DBDC method, the lower and upper bound would be US$100 and US$200, respectively, while in the DBDC-OE method, the lower bound remains the same but the upper bound would be US$250.

As a result, the DBDC-OE approach enhances the accuracy of WTP estimates compared to the traditional DBDC method. Several previous studies have also claimed that the DBDC-OE approach has a smaller degree of starting point bias and incentive incompatibility compared to DBDC alone [18, 19].

Interval regression is used to model outcomes that have interval censoring [20]. Because the data on WTP in this study was developed by the combination of censored and uncensored data, multivariate interval regression was employed to estimate the WTP for RHS. The interval regression method can provide an estimation of the probability of the latent variable located within a certain range. As a result, the dependent variables used in the interval model included both upper-bound and lower-bound variables. These results were then used to determine the average amount customers were willing to pay for RHS.

Multiple logistic regression was used to identify factors associated with the willingness or unwillingness to pay for RHS. Furthermore, based on the contingent valuation method, we used Multiple interval regression to explore factors related to the amount that participants were willing to pay for RHS. A forward stepwise regression with a threshold of *p* < 0.2 was applied to shorten the regression model. A *p*-value < 0.05 was considered statistically significant.

### Ethics approval and consent to participate

All procedures performed in studies involving human participants were in accordance with the ethical standards of the IRB committee at Hanoi Medical University and the Ministry of Health, Vietnam. This research had been performed in accordance with the Helsinki Declaration and its later amendments or comparable ethical standards. Informed consent was obtained from all participants. The study was not conducted on participants under 18 years of age. Before participating in the study, the research subjects clearly explained the meaning of reproductive health services as well as the research purpose.

## Results

Table 1 demonstrates the demographics of the 883 participants. The average age was 41.7 (SD = 11.9) years. Most participants were female (58.8%), and the vast majority had health insurance (74.2%) and were living with their partners (89.6%). The 25.8% who did not have either private or public health insurance paid out of pocket for their medical costs. Overall, 59.1% of participants were willing to pay for RHS. There were significant differences in WTP for RHS across all demographics including age group, sex, education level, marital status, occupation, health insurance status, and household income quintile.

**Table 1:** Demographic characteristics of participants (*n* = *883*)

Characteristics	Not willing to pay ^*(a)*^		Willing to pay ^*(a)*^		Total ^*(b)*^		*p*-value
	***n***	**%**	***n***	**%**	***n***	**%**	
**Total**	361	40.9	522	59.1	883	100.0	
** Age group**							
Young adults (18–30)	63	37.5	105	62.5	168	19.0	< 0.01
Middle-aged adults (31–45)	107	33.8	210	66.3	317	35.9	
Older adults (> 45)	191	48.0	207	52.0	398	45.1	
** Gender**							
Male	193	53.2	170	46.8	363	41.3	< 0.01
Female	167	32.3	350	67.7	517	58.8	
** Education level**							
< High school	135	43.7	174	56.3	309	35.1	< 0.01
High school	114	46.3	132	53.7	246	27.9	
> High school	112	34.4	214	65.6	326	37.0	
** Marital status**							
Single	50	55.6	40	44.4	90	10.4	< 0.01
Living with partner	303	39.1	473	61.0	776	89.6	
** Occupation**							
White collar	43	24.0	136	76.0	179	20.3	< 0.01
Freelancer	78	34.8	146	65.2	224	25.4	
Worker	41	35.0	76	65.0	117	13.3	
Farmer	124	52.1	114	47.9	238	27.0	
Student	14	51.9	13	48.2	27	3.1	
Other	61	62.2	37	37.8	98	11.1	
** Health insurance status**							
Yes	244	37.6	405	62.4	649	74.2	< 0.01
No	114	50.4	112	49.6	226	25.8	
** Household income quintile**							
Quintile 1 (Lowest)	101	48.8	106	51.2	207	23.7	< 0.01
Quintile 2	69	45.7	82	54.3	151	17.3	
Quintile 3	91	41.2	130	58.8	221	25.3	
Quintile 4	48	35.8	86	64.2	134	15.4	
Quintile 5 (Highest)	49	30.6	111	69.4	160	18.3	

*Note:* ^(a)^*percentage calculated by row,* ^(b)^ *percentage calculated by column*

Table 2 describes the health status and health behaviors of participants. Most participants (70.9%) had an underweight BMI (< 18.5 kg/m^2^). Approximately half of the participants (48.5%) reported at least one chronic disease. In terms of health behaviors, 80.7% of participants did not report tobacco use, and 79.2% had one sex partner. Most participants believed that RHS is necessary (69.8%), desired RHS (59.7%), and have used RHS in the past (53.6%). Factors significantly associated with WTP for RHS were number of sex partners, belief in the necessity of RHS, desirability of RHS, and prior use of RHS.

**Table 2:** Health status and health behaviors of participants (*n* = 883)

Health status	Not willing to pay ^*(a)*^		Willing to pay ^*(a)*^		Total ^*(b)*^		*p*-value
	**n**	**%**	**n**	**%**	**n**	**%**	
**BMI**							
Underweight	255	40.7	371	59.3	626	70.9	0.69
Normal	28	45.9	33	54.1	61	6.9	
Overweight/ Obesity	78	39.8	118	60.2	196	22.2	
**Number of chronic diseases**							
0	156	41.5	220	58.5	376	42.9	0.90
1	174	40.9	251	59.1	425	48.5	
≥ 2	29	38.7	46	61.3	75	8.6	
**Current tobacco use**							
No	256	39.6	390	60.4	646	80.7	0.93
Yes	62	40.0	93	60.0	155	19.4	
**Number of sex partners**							
None	97	69.3	43	30.7	140	17.7	< 0.01
One partner	212	33.9	413	66.1	625	79.2	
≥ 2 partners	10	41.7	14	58.3	24	3.0	
**Necessity of reproductive health care (RHS)**							
Necessary	156	25.3	460	74.7	616	69.8	< 0.01
Neutral	151	76.7	46	23.4	197	22.3	
Unnecessary	54	78.3	15	21.7	69	7.8	
**Desire for RHS**							
Yes	74	14.5	438	85.6	512	59.7	< 0.01
No	278	80.6	67	19.4	345	40.3	
**Have ever used RHS**							
Yes	110	23.3	362	76.7	472	53.6	< 0.01
No	251	61.4	158	38.6	409	46.4	

*Note:* ^(a)^*percentage calculated by row,* ^(b)^ *percentage calculated by column*

**Fig. 2:**
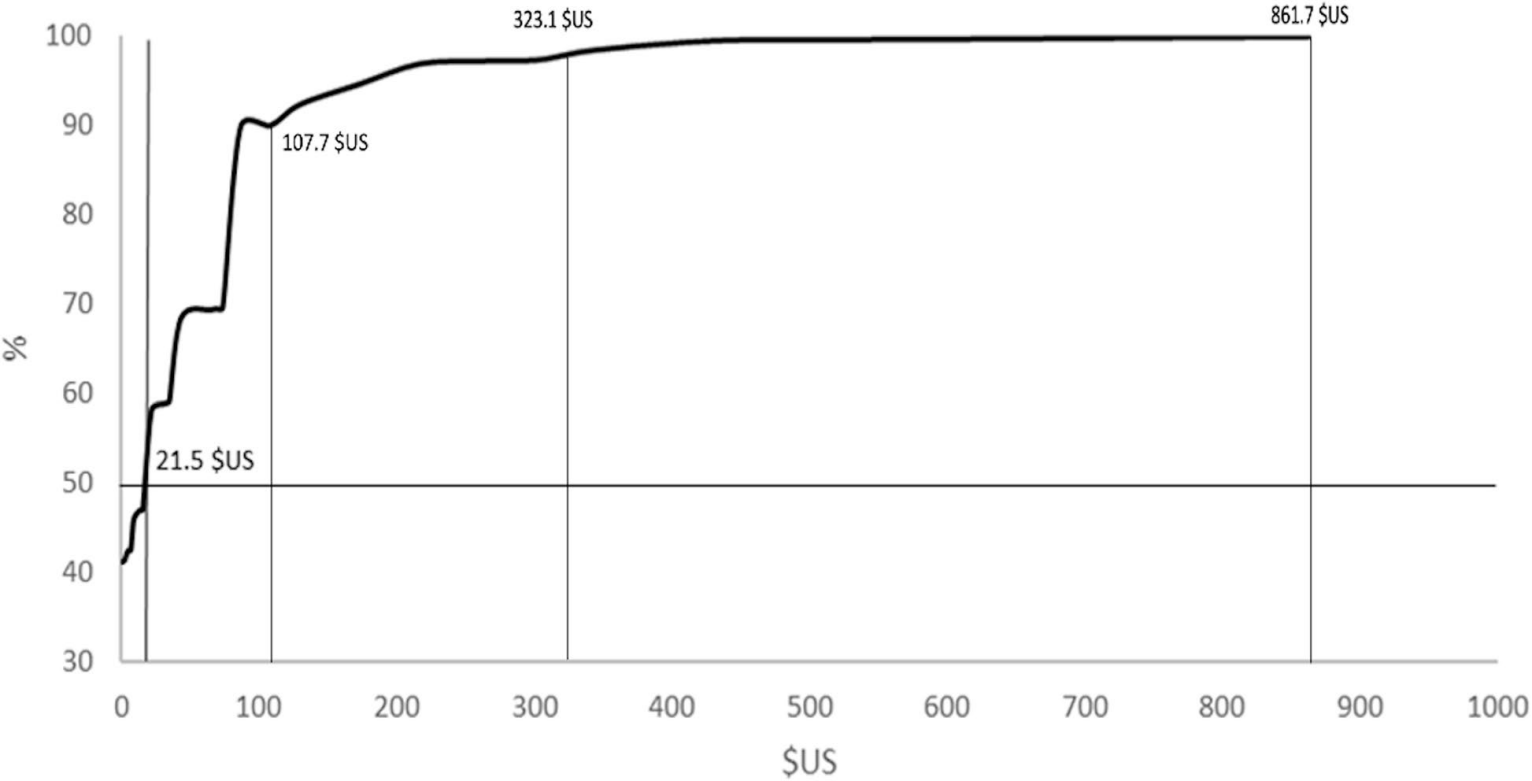
Cumulative proportion of participants willing to pay for RHS by maximum amount

Figure 2 demonstrates that among the 59.1% of subjects willing to pay for RHS, the median maximum amount they were willing to pay was US$21.5. At 107.7$US, only approximately 10% of participants were willing to pay for RHS. With an amount of 323.1 $US or higher, the willingness to pay for RHS is approximately 0%.

Table 3 shows that participants were willing to pay an average of $36.2 ± 2.0 for RHS. Broken.

**Table 3:** Amount willingness to pay for reproductive health care of participants (*n* = 883)

Characteristics	Amount willing to pay (US$)		
	**Mean**	**SD**	**95% CI**
**Total of willing to pay**	$36.2	$2.0	$32.2–$40.2
**Age groups**			
Young Adults	$37.3	$4.4	$28.7–$45.9
Middle-aged Adults	$45.6	$3.9	$38.1–$53.2
Old Adults	$28.2	$2.7	$23.0–$33.4
**Gender**			
Male	$30.7	$3.4	$24.1–$37.3
Female	$40.1	$2.5	$35.2–$45.1
**Education level**			
< High school	$28.6	$2.5	$23.7–$33.5
High school	$30.1	$3.6	$23.0–$37.2
> High school	$47.6	$4.1	$39.6–$55.6
**Marital status**			
Single	$30.9	$7.6	$16.1–$45.7
Living with partner	$37.1	$2.1	$32.9–$41.3
**Occupation**			
White collar	$57.0	$6.0	$45.3–$68.7
Freelancer	$39.1	$3.4	$32.5–$45.6
Worker	$44.0	$5.8	$32.7–$55.4
Farmer	$19.2	$2.8	$13.7–$24.8
Student	$40.7	$18.6	$4.3–$77.1
Other	$22.0	$4.7	$12.8–$31.1
**Health insurance status**			
Yes	$38.0	$2.4	$33.2–$42.7
No	$31.6	$3.8	$24.1–$39.0
**Household income quintile**			
Quintile 1 (Lowest)	$24.3	$3.5	$17.5–$31.2
Quintile 2	$26.8	$3.6	$19.7–$33.9
Quintile 3	$35.1	$3.9	$27.5–$42.7
Quintile 4	$35.2	$3.6	$28.2–$42.1
Quintile 5 (Highest)	$64.0	$7.3	$49.8–$78.3
**BMI**			
Underweight	$30.3	$7.6	$15.4–$45.2
Normal	$39.5	$5.7	$28.4–$50.7
Overweight/ Obesity	$12.8	$2.6	$7.6–$18.0
**Number of chronic diseases**			
0	$37.2	$3.0	$31.3–$43.0
1	$34.4	$2.9	$28.7–$40.2
≥ 2	$41.2	$8.6	$24.3–$58.1
**Current tobacco use**			
No	$38.8	$2.5	$33.8–$43.8
Yes	$32.3	$4.2	$24.0–$40.6
**Number of sex partners**			
None	$18.8	$3.5	$11.9–$25.6
One partner	$37.3	$2.3	$32.8–$41.8
≥ 2 partners	$52.8	$15.5	$22.4–$83.2
**Necessity of RHS**			
Necessary	$49.1	$2.7	$43.7–$54.4
Neutral	$7.4	$1.6	$4.2–$10.5
Unnecessary	$5.6	$1.9	$1.9–$9.4
**Desire for RHS**			
Yes	$51.8	$2.8	$46.4–$57.2
No	$12.8	$2.5	$7.8–$17.8
**Have ever used RHS**			
Yes	$49.9	$2.9	$44.2–$55.6
No	$20.6	$2.6	$15.5–$25.7

down demographically, those with an education level above high school and from the highest income quintile were willing to pay the most in their groups at an average of $47.6 ± 8.0 and $64.0 ± 14.3, respectively. Conversely, farmers and uninsured individuals were willing to pay the least at an average of $19.2 ± 5.6 and $31.6 ± 7.5. Those who believed in the necessity of RHS, desired RHS, and had prior use of RHS were also willing to pay at a higher mean amount—$49.1, $51.8, and $49.9, respectively.

Table 4 shows that the odds of willingness to pay for RHS was lower among the freelancers (OR = 0.44; 95% CI = 0.20; 0.98) and farmers (OR = 0.23; 95%CI = 0.10; 0.49) than those in white-collar jobs. Tobacco use was a significant predictor of WTP for RHS when those had higher odds of willingness to pay for RHS (OR = 3.50; 95%CI = 1.83; 6.69). Participants who were absent of sex partners (OR = 0.39; 95% CI = 0.20; 0.76) had lower odds of WTP for RHS than those with one sex partner. Furthermore, compared to people indicating that the RHS was necessary, the odds of WTP for RHS among people believing RHS was unnecessary (OR = 0.22; 95% CI = 0.09; 0.55) or neutral (OR = 0.20; 95% CI = 0.11; 0.36) was significantly lower. Similarly, the odds of willingness to pay among people who did not have any demand to use RHS (OR = 0.05; 95% CI = 0.03; 0.10), or had never ever used RHS (OR = 0.58; 95% CI = 0.31; 1.09) was significantly lower than the other participants.

**Table 4:** Willingness to pay (WTP) for reproductive health services (*n* = 853)

Characteristics	Willing to pay (Yes vs No)		Amount willing to pay (US$)	
	**OR**	**95% CI**	**Coef**	**95% CI**
**Demographic characteristics**				
** Age groups**				
Young Adults			Ref	
Middle-aged Adults			13.45**	1.27; 25.64
Old Adults			4.28	-8.25; 16.82
** Gender**				
Male			Ref	
Female			-10.13**	-19.76; -0.50
** Occupation**				
White collar	Ref		Ref	
Freelancer	0.44**	0.20; 0.98	-13.13**	-25.27; -0.99
Worker	0.60	0.25; 1.43	-11.69*	-25.20; 1.81
Farmer	0.23***	0.10; 0.49	-26.98***	-39.28; -14.68
Student	1.11	0.04; 28.34	-11.61	-69.32; 46.11
Other	0.20***	0.07; 0.53	-26.95***	-42.83; -11.07
** Having health insurance**				
Yes			Ref	
No			6.30	-3.25; 15.84
** Household income quintiles**				
Quintile 1 (Lowest)			Ref	
Quintile 2			-7.58	-20.03; 4.87
Quintile 3			2.80	-8.68; 14.28
Quintile 4			-0.22	-13.37; 12.94
Quintile 5 (Highest)			29.30***	16.75; 41.85
**Health status and health behaviors**				
** BMI**				
Normal	Ref			
Underweight	0.40*	0.15; 1.02		
Overweight/ Obesity	0.83	0.46; 1.52		
** Current tobacco use**				
No	Ref			
Yes	3.50***	1.83; 6.69		
** Number of sex partners**				
One partner	Ref		Ref	
None	0.39***	0.20; 0.76	-3.24	-14.98; 8.49
≥ 2 partners	2.96*	0.96; 9.14	34.74***	11.56; 57.92
**Demand for RHS**				
** The necessity of RHS**				
Necessary	Ref		Ref	
Neutral	0.20***	0.11; 0.36	-28.09***	-38.98; -17.19
Unnecessary	0.22***	0.09; 0.55	-23.25***	-38.77; -7.72
** Desire for RHS**				
Yes	Ref		Ref	
No	0.05***	0.03; 0.10	-16.95***	-28.54; -5.37
** Have ever used RHS**				
Yes	Ref		Ref	
No	0.58*	0.31; 1.09	-17.43***	-28.03; -6.83

^***^ p < 0.01, ** p < 0.05, * p < *0.1*

The findings also indicate that the amount willing to pay for RHS among people in middle-aged adults (Coef. = 13.45; 95% CI = 1.27; 25.64), those in the highest income quintile (Coef. = 29.30; 95% CI = 16.75; 41.85), and those with two or more sex partners (Coef. = 34.74; 95% CI = 11.56; 57.92) was significantly higher than those in other groups. Meanwhile, compared to males, participants who were females (Coef. = -10.13; 95% CI = -19.76; -0.50) were less amount willing to pay for RHS. Furthermore, the amount of willingness to pay for RHS was significantly lower among freelancers (Coef. = -13.13; 95% CI = -25.27; -0.99) and farmers (Coef. = -26.98; 95% CI = -39.28; -14.68) than their white-collar counterparts. Compared to people who believed that the RHS was necessary, the amount of WTP for RHS among people believing RHS was unnecessary (Coef. = -23.25; 95% CI = -38.77; -7.72) or neutral (Coef. = -28.09; 95% CI = -38.98; -17.19) was significantly lower. Moreover, people who did not have any demand to use RHS (Coef. = -16.95; 95% -28.54; -5.37) or had never ever used RHS (Coef. = -17.43; 95% CI = -28.03; -6.83) were also less amount of WTP for RHS than other participants.

## Discussion

Overall, this study found a moderate level of WTP among adults in a rural district of Hanoi, with 59.1% of participants willing to pay for RHS at an average maximum amount of US$36.2, significantly less than the current average price of US$86.2. This is nearly half of the average individual’s monthly income in Vietnam and a significant hardship for those in lower income quintiles [21]. Health insurance coverage for RHS varies though most patients are required to pay for a portion of their services. The percentage willing to pay is substantially less than the 86.6% and 80.8% found in previous studies willing to pay for HPV and HBV vaccinations, respectively [12, 13]. Though this may be partially explained by differences in study populations (the prior studies were performed among women of reproductive age), the proportion willing to pay remained low even after we adjusted for sex and age.

The low percentage of participants willing to pay for RHS is likely related to an overall low awareness of the necessity of RHS. Indeed, the relationship between knowledge of health services and WTP for those services has been well-demonstrated in the literature [22, 23]. In Vietnam, when it comes to reproductive tract infections, studies have shown a low level of knowledge in both urban and rural areas [24, 25]. Similarly, a study in Southern Vietnam estimated that only about half of women received adequate ANC, which was directly associated with the level of knowledge about ANC [26]. These data suggest persistent gaps in knowledge about RHS among the general population and the need for educational campaigns to increase public awareness and uptake of these services. Indeed, this study found a significant relationship between knowledge, prior use, and belief in the necessity of RHS and WTP for those services, suggesting that improved education could increase WTP with the caveat that services remain affordable for the general population. Other factors that may also contribute to low WTP may be related to issues of access and social norms, which should be explored in future studies.

When the data was broken down by demographics and health behaviors, we found socioeconomic status (SES) and smoking behavior to be strongly associated with WTP for RHS. There were significant differences in WTP for RHS across income quintiles. This suggests the need for both education and financial assistance for RHS, especially among those from lower household income quintiles. Actually, access inequalities in health due to financial restrictions in low-middle-income countries seem to still be a worldwide problem [27]. Hence, our study suggests that subsidized services for low-income people could be a potential solution to achieve equitable access to the RHS. Furthermore, the Government should publicly fund more types of services for reproductive health examination through health insurance. Because, up until now, health insurance in Vietnam has covered above 90% of the population, and health insurance is considered one of the key solutions to solving health inequalities problems [27, 28].

Likewise, white-collar workers demonstrated a higher WTP and were willing to pay a higher amount than freelancers and farmers. The impact of SES on WTP for health services is a trend that has been observed across other low- and middle-income countries [29, 30]. While education level may be a factor mediating these outcomes, these differences persisted even after adjusting for education. Another factor that should also be considered is income predictability, given that white-collar workers tend to have a steadier income than freelancers and farmers. This is a relationship that is underexplored in prior studies on WTP for health services and should be evaluated in future studies.

Notably, another finding of the current study is that tobacco use behavior is associated with a higher WTP for RHS. This finding is understandable when an array of previous studies has demonstrated the strong relationship between smoking behavior and decreased sexual function as well as delay in becoming pregnant or even infertility [31–34]. Hence, our study again suggested that increasing health education programs to control harmful health behaviors as well as improving the accessibility and utilization of RHS for the community, especially vulnerable groups (such as smokers, and alcoholics) can be potential solutions.

The first limitations of this study include its cross-sectional nature, which only allowed us to test for association without insight into causative relationships. Secondly, this study involves self-reporting, which could predispose participants to recall or social desirability bias. Thirdly, the current study has lacked the assessment of some potential factors that could also affect the WTP for RHS such as the number of children, and desire for more children. Hence, further studies could be conducted to explore the association between these factors and the WTP for RHS. Furthermore, because convenience sampling was implemented, this data is not representative of the adult population in Vietnam—further studies should consider collecting data from other urban and rural areas. Moreover, the current study did not distinguish between types of health insurance that are owned by the participants. Therefore, the study's findings may not provide a generalization of the trend as well as the impact of each type of insurance on the willingness to pay decisions differently. Lastly, this study aimed to broadly evaluate WTP for RHS, which included a variety of services from family planning to treatment of sexually transmitted infections. Further studies, should consider examining the type of service, independently, given the heterogeneity of RHS.

## Conclusions

This study found a moderate level of WTP among adults in a rural district of Hanoi with 59.1% of participants willing to pay for RHS at an average maximum amount of US$36.2, significantly less than the current average price of US$86.2. These findings are likely related to an overall low awareness of the necessity of RHS, and future education campaigns should specifically target those from lower-income backgrounds. Financial subsidization should also be considered, especially for those from the low-income group, to ensure equitable access to RHS. Lastly, given the heterogeneity of RHS, further studies should consider examining WTP for each type of service independently.

### Supplementary Information

**Additional file 1:** **Appendix 1. **The number of participants by commune.

## Data Availability

The datasets used and/or analyzed during the current study available from the corresponding author on reasonable request.

## Abbreviations

WTP: Willing to pay
RHS: Reproductive health services
HBV: Hepatitis B virus
HPV: Human papillomavirus
ANC: Antenatal care
SES: Socioeconomic status
