# Supplementary material for: Preference and willingness to pay for reproductive health services among adults in Urban–Rural transition settings of a developing country: evidence from a cross-sectional study in a rural district of Hanoi, Vietnam
Source: BMC Health Serv Res. 2023 Nov 2;23:1196. doi: 10.1186/s12913-023-10207-1 (PMC10623698; doi:10.1186/s12913-023-10207-1)
Supplement: Supplementary file 1 — Additional file 1: Appendix 1. The number of participants by commune. [file 12913_2023_10207_MOESM1_ESM.docx]

**APPENDIX**

**Appendix 1. The number of participants by commune**

| **Communes** | **n** | **%** |
| --- | --- | --- |
| Bich Hoa | 101 | 11.35 |
| Kim An | 100 | 11.24 |
| Lien Chau | 95 | 10.67 |
| Thanh Cao | 100 | 11.24 |
| Thanh Mai | 100 | 11.24 |
| Thanh Van | 100 | 11.24 |
| Kim Bai | 100 | 11.24 |
| Xuan Duong | 94 | 10.56 |
| Tam Hung | 100 | 11.24 |
